# Spatial occurrence of dengue fever and its relationship with land use in Selangor, Malaysia

**DOI:** 10.1186/1471-2458-14-S1-P16

**Published:** 2014-01-29

**Authors:** Haidar Rizal Toha, Jamal Hisham Hashim, Mazrura Sahani, Mohd Shahir Shamsir

**Affiliations:** 1 Jabatan Kesihatan Masyarakat, Pusat Perubatan Universiti Kebangsaan Malaysia, Jalan Yaacob Latif, Bandar Tun Razak, 56000 Cheras, Kuala Lumpur, Malaysia; 2 United Nations University-International Institute for Global Health, UKMMC Complex, Jalan Yaacob Latiff, Bandar Tun Razak, 56000 Cheras, Kuala Lumpur, Malaysia; 3 Faculty of Bioscience and Bioengineering, Universiti Teknologi Malaysia, Skudai, Johor, Malaysia

## Background

Dengue fever has a profound impact in Malaysia, and globally, it has a strong potential to spread to new territories as a consequence of human activities and climate change that modify the environment. Environmental factors can affect the disease epidemic via their influence on vector’s habitat and propagation. Among the economic costs borne in dealing with this matter related to epidemic identification and vector surveillance. The relationship between dengue fever and environmental factors namely land use and degree of urbanisation were investigated in the state of Selangor, Malaysia.

## Materials and methods

Spatial analysis of serologically confirmed dengue cases using Global Moran I and Average Nearest Neighbour methods in four districts, namely Hulu Selangor, Klang, Petaling and Sepang was done to show clustering of cases.

## Results

The clusterings of cases were statistically significant at differing range of distances. Formation of maps of case location, epidemic location, high risk areas and areas that are affected by nearby land uses were done. The land use maps were for agriculture, stagnant water bodies, housing, industry, open land and drainage in all the four districts and the affected areas were shown as hotspots of dengue cases.

## Conclusions

The result of this study is useful for application as a tool to guide health authorities in dengue fever prevention and control activities.

